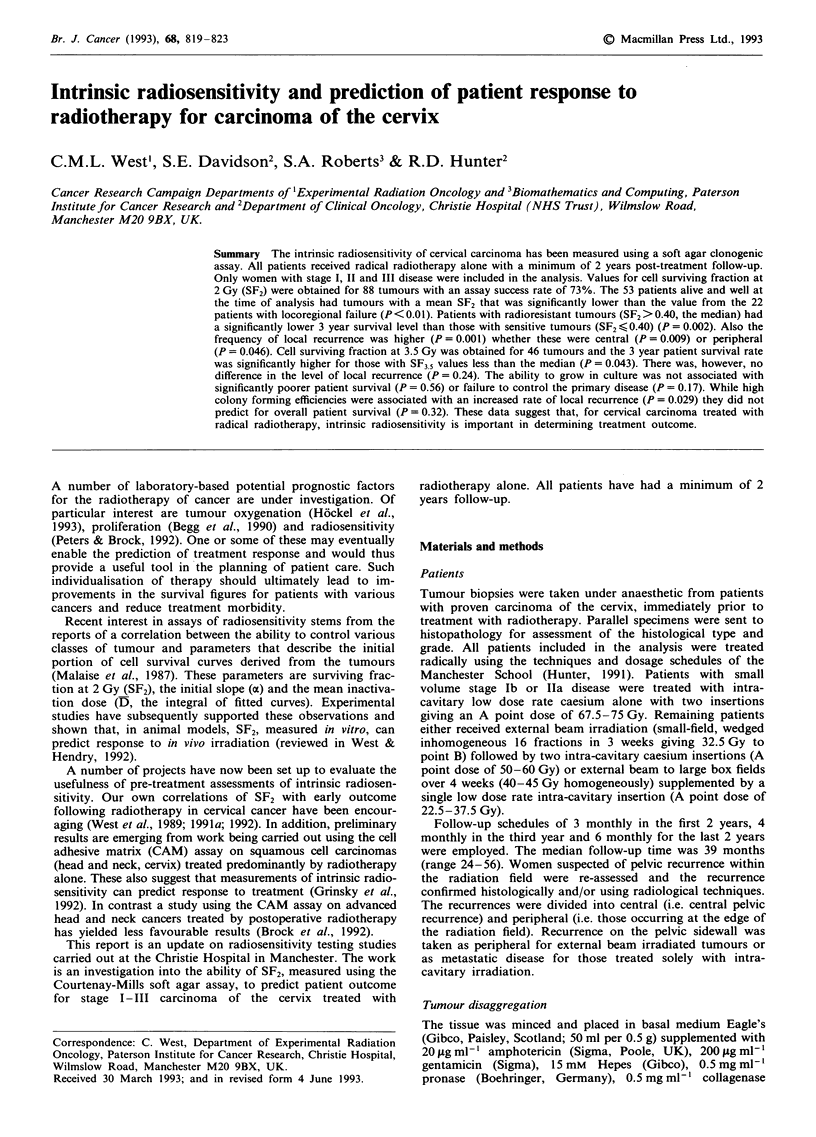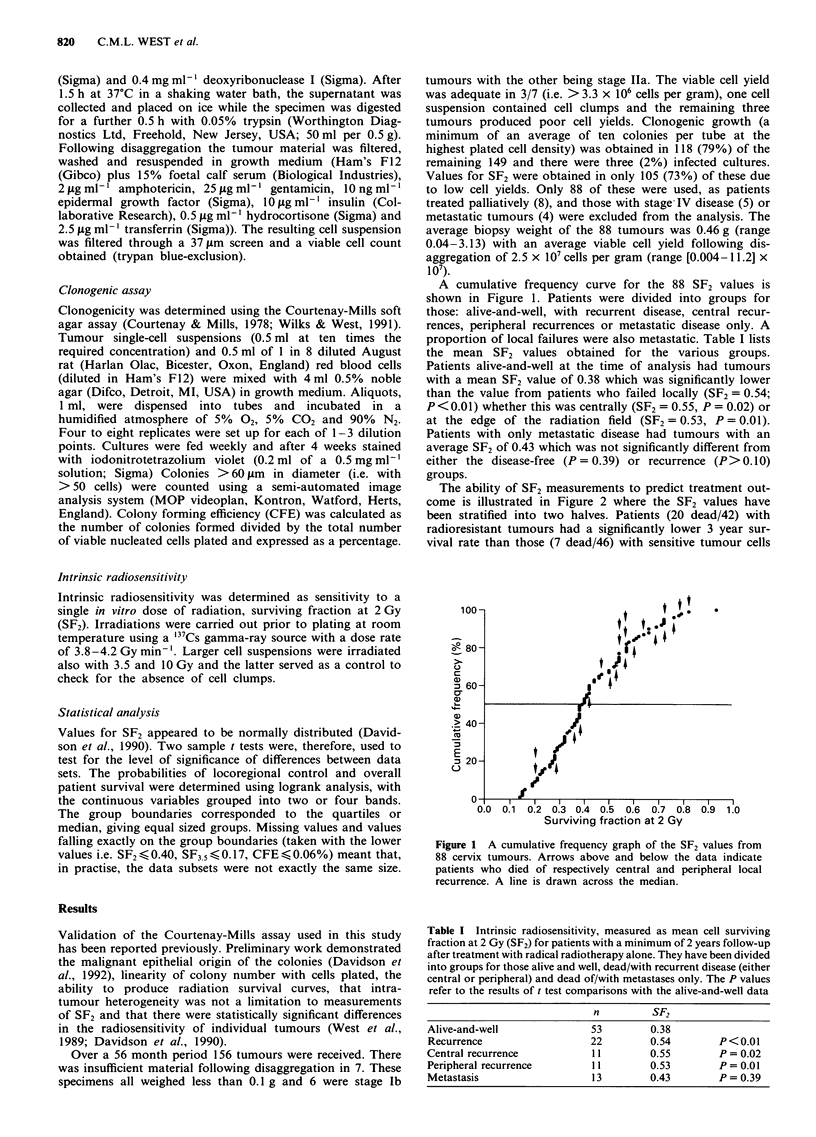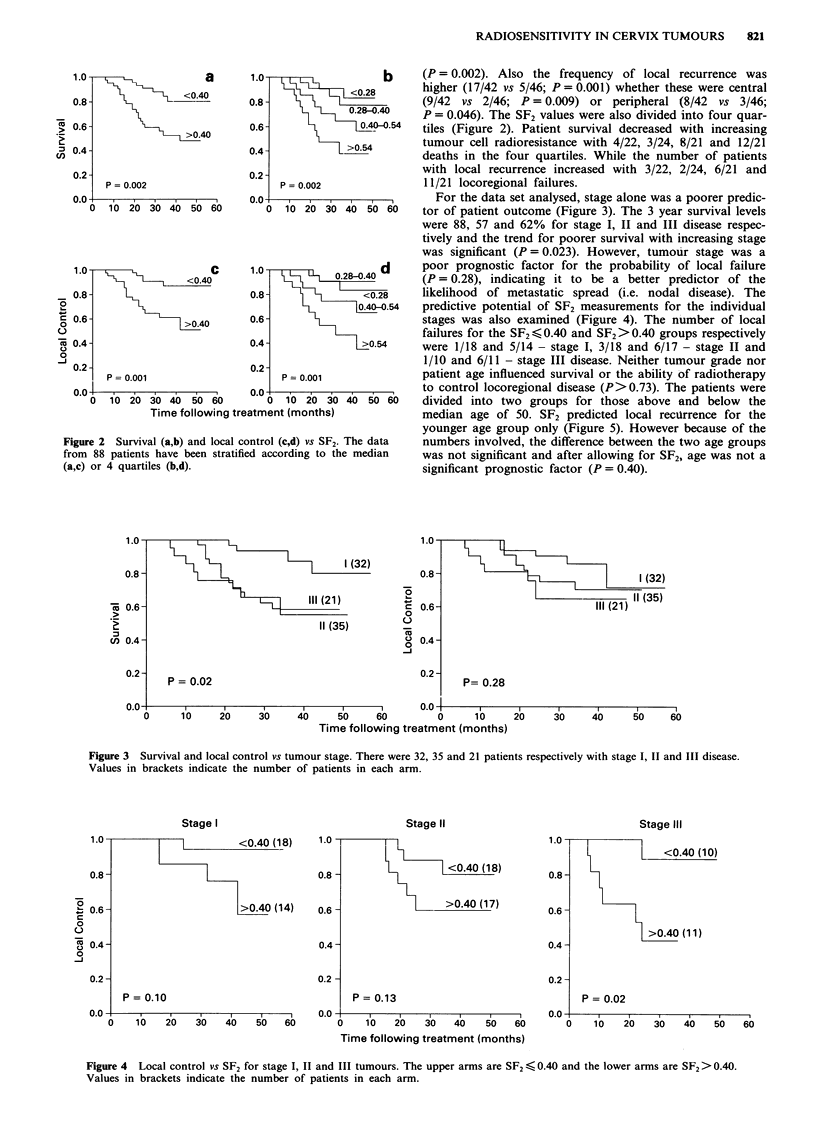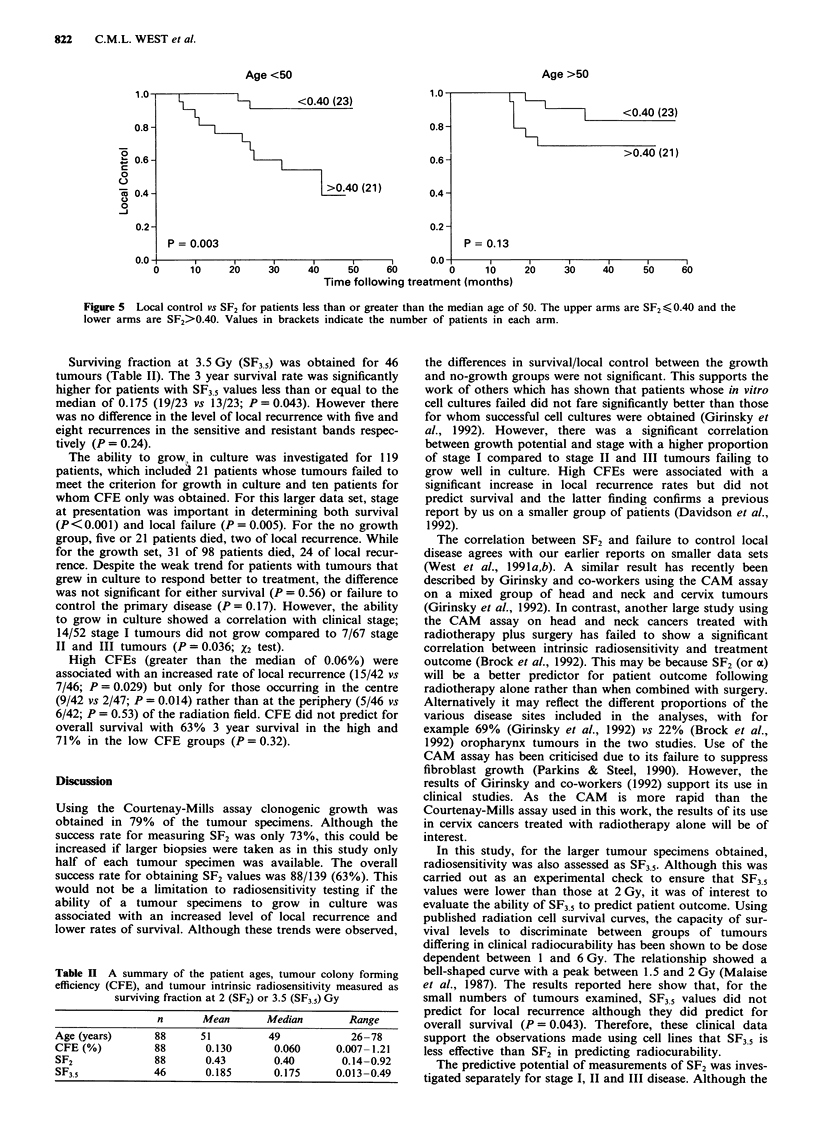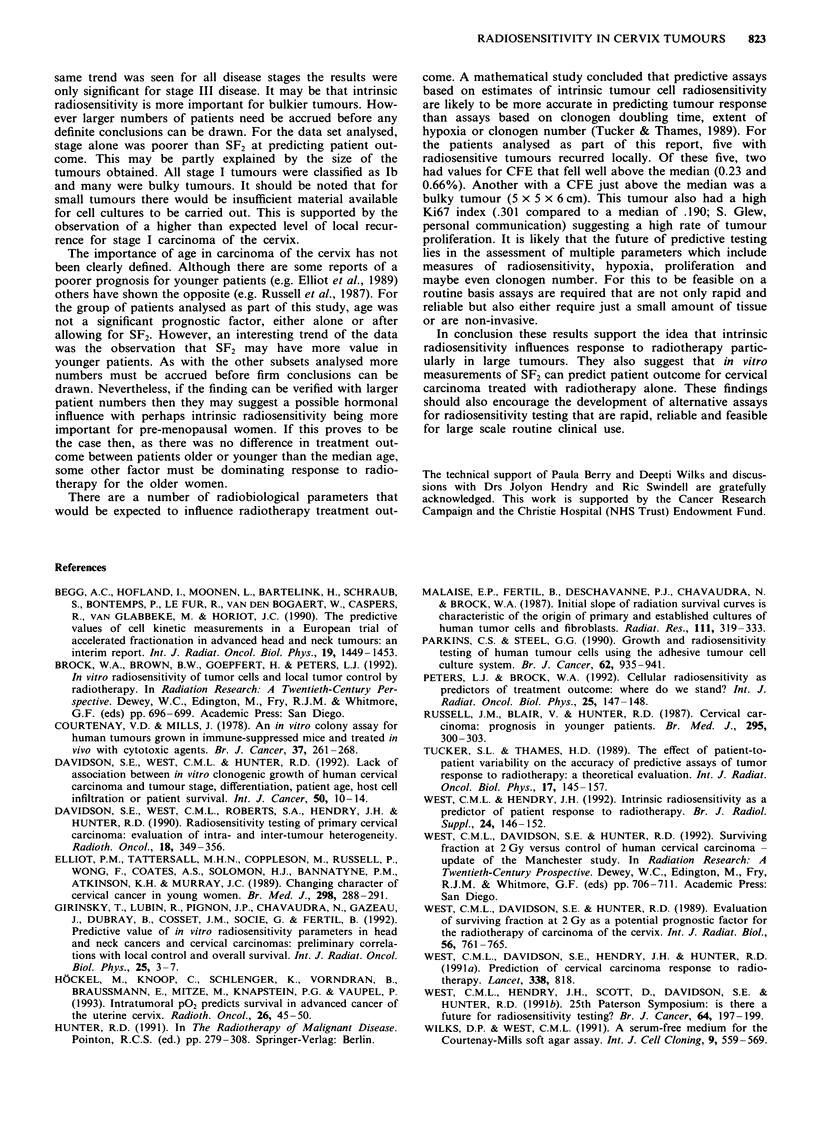# Intrinsic radiosensitivity and prediction of patient response to radiotherapy for carcinoma of the cervix.

**DOI:** 10.1038/bjc.1993.434

**Published:** 1993-10

**Authors:** C. M. West, S. E. Davidson, S. A. Roberts, R. D. Hunter

**Affiliations:** Cancer Research Campaign Department of Experimental Radiation Oncology, Paterson Institute for Cancer Research, Christie Hospital (NHS Trust), Manchester, UK.

## Abstract

The intrinsic radiosensitivity of cervical carcinoma has been measured using a soft agar clonogenic assay. All patients received radical radiotherapy alone with a minimum of 2 years post-treatment follow-up. Only women with stage I, II and III disease were included in the analysis. Values for cell surviving fraction at 2 Gy (SF2) were obtained for 88 tumours with an assay success rate of 73%. The 53 patients alive and well at the time of analysis had tumours with a mean SF2 that was significantly lower than the value from the 22 patients with locoregional failure (P < 0.01). Patients with radioresistant tumours (SF2 > 0.40, the median) had a significantly lower 3 year survival level than those with sensitive tumours (SF2 < or = 0.40) (P = 0.002). Also the frequency of local recurrence was higher (P = 0.001) whether these were central (P = 0.009) or peripheral (P = 0.046). Cell surviving fraction at 3.5 Gy was obtained for 46 tumours and the 3 year patient survival rate was significantly higher for those with SF3.5 values less than the median (P = 0.043). There was, however, no difference in the level of local recurrence (P = 0.24). The ability to grow in culture was not associated with significantly poorer patient survival (P = 0.56) or failure to control the primary disease (P = 0.17). While high colony forming efficiencies were associated with an increased rate of local recurrence (P = 0.029) they did not predict for overall patient survival (P = 0.32). These data suggest that, for cervical carcinoma treated with radical radiotherapy, intrinsic radiosensitivity is important in determining treatment outcome.


Br. J. Cancer (1993), 68, 819-823 © Macmillan Press Ltd., 1993

### Intrinsic radiosensitivity and prediction of patient response to radiotherapy for carcinoma of the cervix

C.M.L. West[1], S.E. Davidson[2], S.A. Roberts[3] & R.D. Hunter[2]

*Cancer Research Campaign Departments of [1]Experimental Radiation Oncology and [3]Biomathematics and Computing, Paterson Institute for Cancer Research and [2]Department of Clinical Oncology, Christie Hospital (NHS Trust), Wilmslow Road, Manchester M20 9BX, UK.*

**Summary** The intrinsic radiosensitivity of cervical carcinoma has been measured using a soft agar clonogenic assay. All patients received radical radiotherapy alone with a minimum of 2 years post-treatment follow-up. Only women with stage I, II and III disease were included in the analysis. Values for cell surviving fraction at 2 Gy ($SF_2$) were obtained for 88 tumours with an assay success rate of 73%. The 53 patients alive and well at the time of analysis had tumours with a mean $SF_2$ that was significantly lower than the value from the 22 patients with locoregional failure ($P < 0.01$). Patients with radioresistant tumours ($SF_2 > 0.40$, the median) had a significantly lower 3 year survival level than those with sensitive tumours ($SF_2 \leqslant 0.40$) ($P = 0.002$). Also the frequency of local recurrence was higher ($P = 0.001$) whether these were central ($P = 0.009$) or peripheral ($P = 0.046$). Cell surviving fraction at 3.5 Gy was obtained for 46 tumours and the 3 year patient survival rate was significantly higher for those with $SF_{3.5}$ values less than the median ($P = 0.043$). There was, however, no difference in the level of local recurrence ($P = 0.24$). The ability to grow in culture was not associated with significantly poorer patient survival ($P = 0.56$) or failure to control the primary disease ($P = 0.17$). While high colony forming efficiencies were associated with an increased rate of local recurrence ($P = 0.029$) they did not predict for overall patient survival ($P = 0.32$). These data suggest that, for cervical carcinoma treated with radical radiotherapy, intrinsic radiosensitivity is important in determining treatment outcome.

A number of laboratory-based potential prognostic factors for the radiotherapy of cancer are under investigation. Of particular interest are tumour oxygenation (Höckel *et al.*, 1993), proliferation (Begg *et al.*, 1990) and radiosensitivity (Peters & Brock, 1992). One or some of these may eventually enable the prediction of treatment response and would thus provide a useful tool in the planning of patient care. Such individualisation of therapy should ultimately lead to improvements in the survival figures for patients with various cancers and reduce treatment morbidity.

Recent interest in assays of radiosensitivity stems from the reports of a correlation between the ability to control various classes of tumour and parameters that describe the initial portion of cell survival curves derived from the tumours (Malaise *et al.*, 1987). These parameters are surviving fraction at 2 Gy ($SF_2$), the initial slope ($\alpha$) and the mean inactivation dose ($\bar{D}$, the integral of fitted curves). Experimental studies have subsequently supported these observations and shown that, in animal models, $SF_2$, measured *in vitro*, can predict response to *in vivo* irradiation (reviewed in West & Hendry, 1992).

A number of projects have now been set up to evaluate the usefulness of pre-treatment assessments of intrinsic radiosensitivity. Our own correlations of $SF_2$ with early outcome following radiotherapy in cervical cancer have been encouraging (West *et al.*, 1989; 1991*a*; 1992). In addition, preliminary results are emerging from work being carried out using the cell adhesive matrix (CAM) assay on squamous cell carcinomas (head and neck, cervix) treated predominantly by radiotherapy alone. These also suggest that measurements of intrinsic radiosensitivity can predict response to treatment (Grinsky *et al.*, 1992). In contrast a study using the CAM assay on advanced head and neck cancers treated by postoperative radiotherapy has yielded less favourable results (Brock *et al.*, 1992).

This report is an update on radiosensitivity testing studies carried out at the Christie Hospital in Manchester. The work is an investigation into the ability of $SF_2$, measured using the Courtenay-Mills soft agar assay, to predict patient outcome for stage I-III carcinoma of the cervix treated with radiotherapy alone. All patients have had a minimum of 2 years follow-up.

Correspondence: C. West, Department of Experimental Radiation Oncology, Paterson Institute for Cancer Research, Christie Hospital, Wilmslow Road, Manchester M20 9BX, UK.
Received 30 March 1993; and in revised form 4 June 1993.

### Materials and methods

### *Patients*

Tumour biopsies were taken under anaesthetic from patients with proven carcinoma of the cervix, immediately prior to treatment with radiotherapy. Parallel specimens were sent to histopathology for assessment of the histological type and grade. All patients included in the analysis were treated radically using the techniques and dosage schedules of the Manchester School (Hunter, 1991). Patients with small volume stage Ib or IIa disease were treated with intracavitary low dose rate caesium alone with two insertions giving an A point dose of 67.5-75 Gy. Remaining patients either received external beam irradiation (small-field, wedged inhomogeneous 16 fractions in 3 weeks giving 32.5 Gy to point B) followed by two intra-cavitary caesium insertions (A point dose of 50-60 Gy) or external beam to large box fields over 4 weeks (40-45 Gy homogeneously) supplemented by a single low dose rate intra-cavitary insertion (A point dose of 22.5-37.5 Gy).

Follow-up schedules of 3 monthly in the first 2 years, 4 monthly in the third year and 6 monthly for the last 2 years were employed. The median follow-up time was 39 months (range 24-56). Women suspected of pelvic recurrence within the radiation field were re-assessed and the recurrence confirmed histologically and/or using radiological techniques. The recurrences were divided into central (i.e. central pelvic recurrence) and peripheral (i.e. those occurring at the edge of the radiation field). Recurrence on the pelvic sidewall was taken as peripheral for external beam irradiated tumours or as metastatic disease for those treated solely with intracavitary irradiation.

### *Tumour disaggregation*

The tissue was minced and placed in basal medium Eagle's (Gibco, Paisley, Scotland; 50 ml per 0.5 g) supplemented with 20 $\mu g\,ml^{-1}$ amphotericin (Sigma, Poole, UK), 200 $\mu g\,ml^{-1}$ gentamicin (Sigma), 15 mM Hepes (Gibco), 0.5 $mg\,ml^{-1}$ pronase (Boehringer, Germany), 0.5 $mg\,ml^{-1}$ collagenase

(Sigma) and 0.4 mg ml$^{-1}$ deoxyribonuclease I (Sigma). After 1.5 h at 37°C in a shaking water bath, the supernatant was collected and placed on ice while the specimen was digested for a further 0.5 h with 0.05% trypsin (Worthington Diagnostics Ltd, Freehold, New Jersey, USA; 50 ml per 0.5 g). Following disaggregation the tumour material was filtered, washed and resuspended in growth medium (Ham's F12 (Gibco) plus 15% foetal calf serum (Biological Industries), 2 $\mu$g ml$^{-1}$ amphotericin, 25 $\mu$g ml$^{-1}$ gentamicin, 10 ng ml$^{-1}$ epidermal growth factor (Sigma), 10 $\mu$g ml$^{-1}$ insulin (Collaborative Research), 0.5 $\mu$g ml$^{-1}$ hydrocortisone (Sigma) and 2.5 $\mu$g ml$^{-1}$ transferrin (Sigma)). The resulting cell suspension was filtered through a 37 $\mu$m screen and a viable cell count obtained (trypan blue-exclusion).

*Clonogenic assay*

Clonogenicity was determined using the Courtenay-Mills soft agar assay (Courtenay & Mills, 1978; Wilks & West, 1991). Tumour single-cell suspensions (0.5 ml at ten times the required concentration) and 0.5 ml of 1 in 8 diluted August rat (Harlan Olac, Bicester, Oxon, England) red blood cells (diluted in Ham's F12) were mixed with 4 ml 0.5% noble agar (Difco, Detroit, MI, USA) in growth medium. Aliquots, 1 ml, were dispensed into tubes and incubated in a humidified atmosphere of 5% $O_2$, 5% $CO_2$ and 90% $N_2$. Four to eight replicates were set up for each of 1–3 dilution points. Cultures were fed weekly and after 4 weeks stained with iodonitrotetrazolium violet (0.2 ml of a 0.5 mg ml$^{-1}$ solution; Sigma) Colonies $>60\,\mu$m in diameter (i.e. with $>50$ cells) were counted using a semi-automated image analysis system (MOP videoplan, Kontron, Watford, Herts, England). Colony forming efficiency (CFE) was calculated as the number of colonies formed divided by the total number of viable nucleated cells plated and expressed as a percentage.

*Intrinsic radiosensitivity*

Intrinsic radiosensitivity was determined as sensitivity to a single *in vitro* dose of radiation, surviving fraction at 2 Gy ($SF_2$). Irradiations were carried out prior to plating at room temperature using a $^{137}$Cs gamma-ray source with a dose rate of 3.8–4.2 Gy min$^{-1}$. Larger cell suspensions were irradiated also with 3.5 and 10 Gy and the latter served as a control to check for the absence of cell clumps.

*Statistical analysis*

Values for $SF_2$ appeared to be normally distributed (Davidson *et al.*, 1990). Two sample *t* tests were, therefore, used to test for the level of significance of differences between data sets. The probabilities of locoregional control and overall patient survival were determined using logrank analysis, with the continuous variables grouped into two or four bands. The group boundaries corresponded to the quartiles or median, giving equal sized groups. Missing values and values falling exactly on the group boundaries (taken with the lower values i.e. $SF_2 \leqslant 0.40$, $SF_{3.5} \leqslant 0.17$, CFE$\leqslant$0.06%) meant that, in practise, the data subsets were not exactly the same size.

### Results

Validation of the Courtenay-Mills assay used in this study has been reported previously. Preliminary work demonstrated the malignant epithelial origin of the colonies (Davidson *et al.*, 1992), linearity of colony number with cells plated, the ability to produce radiation survival curves, that intratumour heterogeneity was not a limitation to measurements of $SF_2$ and that there were statistically significant differences in the radiosensitivity of individual tumours (West *et al.*, 1989; Davidson *et al.*, 1990).

Over a 56 month period 156 tumours were received. There was insufficient material following disaggregation in 7. These specimens all weighed less than 0.1 g and 6 were stage Ib tumours with the other being stage IIa. The viable cell yield was adequate in 3/7 (i.e. $>3.3\times10^6$ cells per gram), one cell suspension contained cell clumps and the remaining three tumours produced poor cell yields. Clonogenic growth (a minimum of an average of ten colonies per tube at the highest plated cell density) was obtained in 118 (79%) of the remaining 149 and there were three (2%) infected cultures. Values for $SF_2$ were obtained in only 105 (73%) of these due to low cell yields. Only 88 of these were used, as patients treated palliatively (8), and those with stage IV disease (5) or metastatic tumours (4) were excluded from the analysis. The average biopsy weight of the 88 tumours was 0.46 g (range 0.04–3.13) with an average viable cell yield following disaggregation of $2.5\times10^7$ cells per gram (range $[0.004-11.2]\times10^7$).

A cumulative frequency curve for the 88 $SF_2$ values is shown in Figure 1. Patients were divided into groups for those: alive-and-well, with recurrent disease, central recurrences, peripheral recurrences or metastatic disease only. A proportion of local failures were also metastatic. Table I lists the mean $SF_2$ values obtained for the various groups. Patients alive-and-well at the time of analysis had tumours with a mean $SF_2$ value of 0.38 which was significantly lower than the value from patients who failed locally ($SF_2=0.54$; $P<0.01$) whether this was centrally ($SF_2=0.55$, $P=0.02$) or at the edge of the radiation field ($SF_2=0.53$, $P=0.01$). Patients with only metastatic disease had tumours with an average $SF_2$ of 0.43 which was not significantly different from either the disease-free ($P=0.39$) or recurrence ($P>0.10$) groups.

The ability of $SF_2$ measurements to predict treatment outcome is illustrated in Figure 2 where the $SF_2$ values have been stratified into two halves. Patients (20 dead/42) with radioresistant tumours had a significantly lower 3 year survival rate than those (7 dead/46) with sensitive tumour cells

**Figure 1** A cumulative frequency graph of the $SF_2$ values from 88 cervix tumours. Arrows above and below the data indicate patients who died of respectively central and peripheral local recurrence. A line is drawn across the median.

**Table I** Intrinsic radiosensitivity, measured as mean cell surviving fraction at 2 Gy ($SF_2$) for patients with a minimum of 2 years follow-up after treatment with radical radiotherapy alone. They have been divided into groups for those alive and well, dead/with recurrent disease (either central or peripheral) and dead of/with metastases only. The *P* values refer to the results of *t* test comparisons with the alive-and-well data

| | *n* | $SF_2$ | |
|---|---|---|---|
| Alive-and-well | 53 | 0.38 | |
| Recurrence | 22 | 0.54 | $P<0.01$ |
| Central recurrence | 11 | 0.55 | $P=0.02$ |
| Peripheral recurrence | 11 | 0.53 | $P=0.01$ |
| Metastasis | 13 | 0.43 | $P=0.39$ |

**Figure 2** Survival (**a**,**b**) and local control (**c**,**d**) *vs* $SF_2$. The data from 88 patients have been stratified according to the median (**a**,**c**) or 4 quartiles (**b**,**d**).

($P = 0.002$). Also the frequency of local recurrence was higher (17/42 *vs* 5/46; $P = 0.001$) whether these were central (9/42 *vs* 2/46; $P = 0.009$) or peripheral (8/42 *vs* 3/46; $P = 0.046$). The $SF_2$ values were also divided into four quartiles (Figure 2). Patient survival decreased with increasing tumour cell radioresistance with 4/22, 3/24, 8/21 and 12/21 deaths in the four quartiles. While the number of patients with local recurrence increased with 3/22, 2/24, 6/21 and 11/21 locoregional failures.

For the data set analysed, stage alone was a poorer predictor of patient outcome (Figure 3). The 3 year survival levels were 88, 57 and 62% for stage I, II and III disease respectively and the trend for poorer survival with increasing stage was significant ($P = 0.023$). However, tumour stage was a poor prognostic factor for the probability of local failure ($P = 0.28$), indicating it to be a better predictor of the likelihood of metastatic spread (i.e. nodal disease). The predictive potential of $SF_2$ measurements for the individual stages was also examined (Figure 4). The number of local failures for the $SF_2 \leqslant 0.40$ and $SF_2 > 0.40$ groups respectively were 1/18 and 5/14 – stage I, 3/18 and 6/17 – stage II and 1/10 and 6/11 – stage III disease. Neither tumour grade nor patient age influenced survival or the ability of radiotherapy to control locoregional disease ($P > 0.73$). The patients were divided into two groups for those above and below the median age of 50. $SF_2$ predicted local recurrence for the younger age group only (Figure 5). However because of the numbers involved, the difference between the two age groups was not significant and after allowing for $SF_2$, age was not a significant prognostic factor ($P = 0.40$).

**Figure 3** Survival and local control *vs* tumour stage. There were 32, 35 and 21 patients respectively with stage I, II and III disease. Values in brackets indicate the number of patients in each arm.

**Figure 4** Local control *vs* $SF_2$ for stage I, II and III tumours. The upper arms are $SF_2 \leqslant 0.40$ and the lower arms are $SF_2 > 0.40$. Values in brackets indicate the number of patients in each arm.

**Figure 5** Local control vs $SF_2$ for patients less than or greater than the median age of 50. The upper arms are $SF_2 \leq 0.40$ and the lower arms are $SF_2 > 0.40$. Values in brackets indicate the number of patients in each arm.

Surviving fraction at 3.5 Gy ($SF_{3.5}$) was obtained for 46 tumours (Table II). The 3 year survival rate was significantly higher for patients with $SF_{3.5}$ values less than or equal to the median of 0.175 (19/23 vs 13/23; $P = 0.043$). However there was no difference in the level of local recurrence with five and eight recurrences in the sensitive and resistant bands respectively ($P = 0.24$).

The ability to grow in culture was investigated for 119 patients, which included 21 patients whose tumours failed to meet the criterion for growth in culture and ten patients for whom CFE only was obtained. For this larger data set, stage at presentation was important in determining both survival ($P < 0.001$) and local failure ($P = 0.005$). For the no growth group, five or 21 patients died, two of local recurrence. While for the growth set, 31 of 98 patients died, 24 of local recurrence. Despite the weak trend for patients with tumours that grew in culture to respond better to treatment, the difference was not significant for either survival ($P = 0.56$) or failure to control the primary disease ($P = 0.17$). However, the ability to grow in culture showed a correlation with clinical stage; 14/52 stage I tumours did not grow compared to 7/67 stage II and III tumours ($P = 0.036$; $\chi_2$ test).

High CFEs (greater than the median of 0.06%) were associated with an increased rate of local recurrence (15/42 vs 7/46; $P = 0.029$) but only for those occurring in the centre (9/42 vs 2/47; $P = 0.014$) rather than at the periphery (5/46 vs 6/42; $P = 0.53$) of the radiation field. CFE did not predict for overall survival with 63% 3 year survival in the high and 71% in the low CFE groups ($P = 0.32$).

### Discussion

Using the Courtenay-Mills assay clonogenic growth was obtained in 79% of the tumour specimens. Although the success rate for measuring $SF_2$ was only 73%, this could be increased if larger biopsies were taken as in this study only half of each tumour specimen was available. The overall success rate for obtaining $SF_2$ values was 88/139 (63%). This would not be a limitation to radiosensitivity testing if the ability of a tumour specimens to grow in culture was associated with an increased level of local recurrence and lower rates of survival. Although these trends were observed, the differences in survival/local control between the growth and no-growth groups were not significant. This supports the work of others which has shown that patients whose *in vitro* cell cultures failed did not fare significantly better than those for whom successful cell cultures were obtained (Girinsky *et al.*, 1992). However, there was a significant correlation between growth potential and stage with a higher proportion of stage I compared to stage II and III tumours failing to grow well in culture. High CFEs were associated with a significant increase in local recurrence rates but did not predict survival and the latter finding confirms a previous report by us on a smaller group of patients (Davidson *et al.*, 1992).

**Table II** A summary of the patient ages, tumour colony forming efficiency (CFE), and tumour intrinsic radiosensitivity measured as surviving fraction at 2 ($SF_2$) or 3.5 ($SF_{3.5}$) Gy

| | *n* | *Mean* | *Median* | *Range* |
|---|---|---|---|---|
| Age (years) | 88 | 51 | 49 | 26–78 |
| CFE (%) | 88 | 0.130 | 0.060 | 0.007–1.21 |
| $SF_2$ | 88 | 0.43 | 0.40 | 0.14–0.92 |
| $SF_{3.5}$ | 46 | 0.185 | 0.175 | 0.013–0.49 |

The correlation between $SF_2$ and failure to control local disease agrees with our earlier reports on smaller data sets (West *et al.*, 1991*a*,*b*). A similar result has recently been described by Girinsky and co-workers using the CAM assay on a mixed group of head and neck and cervix tumours (Girinsky *et al.*, 1992). In contrast, another large study using the CAM assay on head and neck cancers treated with radiotherapy plus surgery has failed to show a significant correlation between intrinsic radiosensitivity and treatment outcome (Brock *et al.*, 1992). This may be because $SF_2$ (or $\alpha$) will be a better predictor for patient outcome following radiotherapy alone rather than when combined with surgery. Alternatively it may reflect the different proportions of the various disease sites included in the analyses, with for example 69% (Girinsky *et al.*, 1992) vs 22% (Brock *et al.*, 1992) oropharynx tumours in the two studies. Use of the CAM assay has been criticised due to its failure to suppress fibroblast growth (Parkins & Steel, 1990). However, the results of Girinsky and co-workers (1992) support its use in clinical studies. As the CAM is more rapid than the Courtenay-Mills assay used in this work, the results of its use in cervix cancers treated with radiotherapy alone will be of interest.

In this study, for the larger tumour specimens obtained, radiosensitivity was also assessed as $SF_{3.5}$. Although this was carried out as an experimental check to ensure that $SF_{3.5}$ values were lower than those at 2 Gy, it was of interest to evaluate the ability of $SF_{3.5}$ to predict patient outcome. Using published radiation cell survival curves, the capacity of survival levels to discriminate between groups of tumours differing in clinical radiocurability has been shown to be dose dependent between 1 and 6 Gy. The relationship showed a bell-shaped curve with a peak between 1.5 and 2 Gy (Malaise *et al.*, 1987). The results reported here show that, for the small numbers of tumours examined, $SF_{3.5}$ values did not predict for local recurrence although they did predict for overall survival ($P = 0.043$). Therefore, these clinical data support the observations made using cell lines that $SF_{3.5}$ is less effective than $SF_2$ in predicting radiocurability.

The predictive potential of measurements of $SF_2$ was investigated separately for stage I, II and III disease. Although the same trend was seen for all disease stages the results were only significant for stage III disease. It may be that intrinsic radiosensitivity is more important for bulkier tumours. However larger numbers of patients need be accrued before any definite conclusions can be drawn. For the data set analysed, stage alone was poorer than $SF_2$ at predicting patient outcome. This may be partly explained by the size of the tumours obtained. All stage I tumours were classified as Ib and many were bulky tumours. It should be noted that for small tumours there would be insufficient material available for cell cultures to be carried out. This is supported by the observation of a higher than expected level of local recurrence for stage I carcinoma of the cervix.

The importance of age in carcinoma of the cervix has not been clearly defined. Although there are some reports of a poorer prognosis for younger patients (e.g. Elliot *et al.*, 1989) others have shown the opposite (e.g. Russell *et al.*, 1987). For the group of patients analysed as part of this study, age was not a significant prognostic factor, either alone or after allowing for $SF_2$. However, an interesting trend of the data was the observation that $SF_2$ may have more value in younger patients. As with the other subsets analysed more numbers must be accrued before firm conclusions can be drawn. Nevertheless, if the finding can be verified with larger patient numbers then they may suggest a possible hormonal influence with perhaps intrinsic radiosensitivity being more important for pre-menopausal women. If this proves to be the case then, as there was no difference in treatment outcome between patients older or younger than the median age, some other factor must be dominating response to radiotherapy for the older women.

There are a number of radiobiological parameters that would be expected to influence radiotherapy treatment outcome. A mathematical study concluded that predictive assays based on estimates of intrinsic tumour cell radiosensitivity are likely to be more accurate in predicting tumour response than assays based on clonogen doubling time, extent of hypoxia or clonogen number (Tucker & Thames, 1989). For the patients analysed as part of this report, five with radiosensitive tumours recurred locally. Of these five, two had values for CFE that fell well above the median (0.23 and 0.66%). Another with a CFE just above the median was a bulky tumour ($5 \times 5 \times 6$ cm). This tumour also had a high Ki67 index (.301 compared to a median of .190; S. Glew, personal communication) suggesting a high rate of tumour proliferation. It is likely that the future of predictive testing lies in the assessment of multiple parameters which include measures of radiosensitivity, hypoxia, proliferation and maybe even clonogen number. For this to be feasible on a routine basis assays are required that are not only rapid and reliable but also either require just a small amount of tissue or are non-invasive.

In conclusion these results support the idea that intrinsic radiosensitivity influences response to radiotherapy particularly in large tumours. They also suggest that *in vitro* measurements of $SF_2$ can predict patient outcome for cervical carcinoma treated with radiotherapy alone. These findings should also encourage the development of alternative assays for radiosensitivity testing that are rapid, reliable and feasible for large scale routine clinical use.

The technical support of Paula Berry and Deepti Wilks and discussions with Drs Jolyon Hendry and Ric Swindell are gratefully acknowledged. This work is supported by the Cancer Research Campaign and the Christie Hospital (NHS Trust) Endowment Fund.

### References

BEGG, A.C., HOFLAND, I., MOONEN, L., BARTELINK, H., SCHRAUB, S., BONTEMPS, P., LE FUR, R., VAN DEN BOGAERT, W., CASPERS, R., VAN GLABBEKE, M. & HORIOT, J.C. (1990). The predictive values of cell kinetic measurements in a European trial of accelerated fractionation in advanced head and neck tumours: an interim report. *Int. J. Radiat. Oncol. Biol. Phys.*, **19**, 1449-1453.

BROCK, W.A., BROWN, B.W., GOEPFERT, H. & PETERS, L.J. (1992). *In vitro* radiosensitivity of tumor cells and local tumor control by radiotherapy. In *Radiation Research: A Twentieth-Century Perspective*. Dewey, W.C., Edington, M., Fry, R.J.M. & Whitmore, G.F. (eds) pp. 696-699. Academic Press: San Diego.

COURTENAY, V.D. & MILLS, J. (1978). An *in vitro* colony assay for human tumours grown in immune-suppressed mice and treated *in vivo* with cytotoxic agents. *Br. J. Cancer*, **37**, 261-268.

DAVIDSON, S.E., WEST, C.M.L. & HUNTER, R.D. (1992). Lack of association between *in vitro* clonogenic growth of human cervical carcinoma and tumour stage, differentiation, patient age, host cell infiltration or patient survival. *Int. J. Cancer*, **50**, 10-14.

DAVIDSON, S.E., WEST, C.M.L., ROBERTS, S.A., HENDRY, J.H. & HUNTER, R.D. (1990). Radiosensitivity testing of primary cervical carcinoma: evaluation of intra- and inter-tumour heterogeneity. *Radioth. Oncol.*, **18**, 349-356.

ELLIOT, P.M., TATTERSALL, M.H.N., COPPLESON, M., RUSSELL, P., WONG, F., COATES, A.S., SOLOMON, H.J., BANNATYNE, P.M., ATKINSON, K.H. & MURRAY, J.C. (1989). Changing character of cervical cancer in young women. *Br. Med. J.*, **298**, 288-291.

GIRINSKY, T., LUBIN, R., PIGNON, J.P., CHAVAUDRA, N., GAZEAU, J., DUBRAY, B., COSSET, J.M., SOCIE, G. & FERTIL, B. (1992). Predictive value of *in vitro* radiosensitivity parameters in head and neck cancers and cervical carcinomas: preliminary correlations with local control and overall survival. *Int. J. Radiat. Oncol. Biol. Phys.*, **25**, 3-7.

HÖCKEL, M., KNOOP, C., SCHLENGER, K., VORNDRAN, B., BRAUSSMANN, E., MITZE, M., KNAPSTEIN, P.G. & VAUPEL, P. (1993). Intratumoral $pO_2$ predicts survival in advanced cancer of the uterine cervix. *Radioth. Oncol.*, **26**, 45-50.

HUNTER, R.D. (1991). In *The Radiotherapy of Malignant Disease*. Pointon, R.C.S. (ed.) pp. 279-308. Springer-Verlag: Berlin.

MALAISE, E.P., FERTIL, B., DESCHAVANNE, P.J., CHAVAUDRA, N. & BROCK, W.A. (1987). Initial slope of radiation survival curves is characteristic of the origin of primary and established cultures of human tumor cells and fibroblasts. *Radiat. Res.*, **111**, 319-333.

PARKINS, C.S. & STEEL, G.G. (1990). Growth and radiosensitivity testing of human tumour cells using the adhesive tumour cell culture system. *Br. J. Cancer*, **62**, 935-941.

PETERS, L.J. & BROCK, W.A. (1992). Cellular radiosensitivity as predictors of treatment outcome: where do we stand? *Int. J. Radiat. Oncol. Biol. Phys.*, **25**, 147-148.

RUSSELL, J.M., BLAIR, V. & HUNTER, R.D. (1987). Cervical carcinoma: prognosis in younger patients. *Br. Med. J.*, **295**, 300-303.

TUCKER, S.L. & THAMES, H.D. (1989). The effect of patient-to-patient variability on the accuracy of predictive assays of tumor response to radiotherapy: a theoretical evaluation. *Int. J. Radiat. Oncol. Biol. Phys.*, **17**, 145-157.

WEST, C.M.L. & HENDRY, J.H. (1992). Intrinsic radiosensitivity as a predictor of patient response to radiotherapy. *Br. J. Radiol. Suppl.*, **24**, 146-152.

WEST, C.M.L., DAVIDSON, S.E. & HUNTER, R.D. (1992). Surviving fraction at 2 Gy versus control of human cervical carcinoma – update of the Manchester study. In *Radiation Research: A Twentieth-Century Prospective*. Dewey, W.C., Edington, M., Fry, R.J.M. & Whitmore, G.F. (eds) pp. 706-711. Academic Press: San Diego.

WEST, C.M.L., DAVIDSON, S.E. & HUNTER, R.D. (1989). Evaluation of surviving fraction at 2 Gy as a potential prognostic factor for the radiotherapy of carcinoma of the cervix. *Int. J. Radiat. Biol.*, **56**, 761-765.

WEST, C.M.L., DAVIDSON, S.E., HENDRY, J.H. & HUNTER, R.D. (1991*a*). Prediction of cervical carcinoma response to radiotherapy. *Lancet*, **338**, 818.

WEST, C.M.L., HENDRY, J.H., SCOTT, D., DAVIDSON, S.E. & HUNTER, R.D. (1991*b*). 25th Paterson Symposium: is there a future for radiosensitivity testing? *Br. J. Cancer*, **64**, 197-199.

WILKS, D.P. & WEST, C.M.L. (1991). A serum-free medium for the Courtenay-Mills soft agar assay. *Int. J. Cell Cloning*, **9**, 559-569.